# Cancer

**Published:** 1886-12

**Authors:** 


### CANCER.

The predisposing causes of cancer are in the habits of the patients themselves. Just as civilization is the cause of the strain that wrecks so many intellects, so it is also the cause of depressing the animal vitality of the individual, and brings in its train this dread disease. The main cause of this disease is "established wealth and a state of luxury." The appetite for eating meat and highly seasoned food is indulged, and can be regulated and habitually indulged, only in a state of established civilization, with communities engaged in accumulating fortunes and vieing with each other in sumptuous living." These conditions, together with habits of indolence and insufficient exercise, cause an accumulation of the waste products in the system which predisposes to cancer. Then, an accidental bruise, or reversal of fortune with mental depression, or any other exciting cause, may develop this terrible disease.

The lesson is obvious. People should live more frugally and take plenty of exercise in the open air, and, in short, follow hygienic modes of living, and the danger of cancer is much more remote. The cure may be difficult, but prevention seems to be in the power of the individual.—*Med. Summary*.

